# Exercise reduces body fat and improves insulin sensitivity and pancreatic β-cell function in overweight and obese male Taiwanese adolescents

**DOI:** 10.1186/s12887-018-1025-y

**Published:** 2018-02-22

**Authors:** Kuang-Chung Shih, Ching-Fai Kwok

**Affiliations:** 1 Division of Endocrinology and Metabolism, Department of Medicine, Cheng-Hsin General Hospital, Taipei, Taiwan; 2 Division of Endocrinology and Metabolism, Tri-Service General Hospital, National Defense Medical Center, Taipei, Taiwan; 3 Faculty of Medicine, School of Medicine, National Yang-Ming University, Taipei, Taiwan; 4 Division of Endocrinology and Metabolism, Department of Medicine, Taipei-Veteran General Hospital, 201, Sec. 2, Shih-Pai Rd, Taipei, 112 Taiwan

**Keywords:** β-cell function, Exercise, Insulin resistance, Obesity, Type 2 diabetes

## Abstract

**Background:**

Improvements in insulin resistance and pancreatic β-cell function have been shown following exercise in adults with obesity; however, few adolescent-based studies have been conducted. This study examined the impact of exercise training on body fat and insulin sensitivity and secretion in overweight and obese adolescents.

**Methods:**

The effects of a 12-week exercise program on the parameters of adiposity and glucose homeostasis were investigated in 47 overweight and obese male adolescents.

**Results:**

After the exercise training program, body weight, body mass index, waist circumference, and body fat were significantly decreased (*P* < 0.001). Improvements in insulin sensitivity (HOMA-IR: 1.40 vs. 0.86, *P* < 0.001) and the disposition index (5.84 vs. 12.77, *P* < 0.001) were also observed. Compared to baseline, oral glucose tolerance tests showed reduced glucose and insulin levels at all time points following the exercise training (all *P* < 0.001). Subgroup analysis of overweight and obese adolescents with abnormal glucose tolerance revealed that there was no difference in plasma glucose levels as compared to the lean group.

**Conclusions:**

A 12-week exercise training is effective in reducing body fat and improving insulin sensitivity and secretion. In addition, the benefits of the exercise intervention were even experienced by those with impaired glucose tolerance.

## Background

Type 2 diabetes mellitus (T2DM) is now increasingly diagnosed in children and adolescents [1–3], which is likely due to the increased prevalence of childhood obesity. High childhood body mass index (BMI) is positively associated with adult coronary heart disease, diabetes and a range of cancers. β-cell dysfunction and insulin resistance are key elements in the pathogenesis of T2DM in both youth and adults patients [4, 5]. It is postulated that in the development of T2DM in youth, an early defect in first-phase insulin response, is followed by defects in second-phase response with development of overt diabetes [6, 7], and these deteriorations occur in the context of a decline in insulin sensitivity. Thus, given its high prevalence and link to chronic disease, early effective interventions for the treatment of childhood obesity and its metabolic abnormalities are urgently needed.

The benefits of exercise for improving glucose homeostasis and insulin resistance are well-established for adults with overweight and obesity, and amelioration of the insulin sensitivity and β-cell function following exercise training is independent of reductions in body weight or adipose tissue as well as improved body lean mass. For example, large improvements in insulin resistance occur after exercise and/or caloric restriction in obese adults with and without diabetes [8, 9]. In addition, physical activities of moderate intensity can substantially reduce the risk of T2DM [10]. Furthermore, previous studies have also highlighted the beneficial effects of lifestyle interventions on insulin secretion and β-cell function in adults with obesity [11–13]. Moreover, a recent meta-analysis that included nine randomized controlled trials reported improvements in insulin resistance markers in youth with obesity with aerobic exercise programs lasting > 12 weeks [14]. In lean male adolescents, we have previously shown that a 12-week exercise program reduced many anthropomorphic measures and blood pressure while increasing the levels of anti-inflammatory markers, such as adiponectin, interleukin-6 (IL-6), and C-reactive protein [15]. In addition to improved β-cell function, the same exercise intervention also improved insulin sensitivity with similar reductions in anthropomorphic measures in male adolescents with obesity [16]. However, to our knowledge, only one study has examined the effects of exercise on β-cell function in adolescents [17].

We sought to test the hypothesis that exercise training would be associated with reductions in body fat and improvements in insulin sensitivity and secretion in overweight and obese Taiwanese male adolescents. In the present study, the effects of a 12-week exercise program on the parameters of adiposity and glucose homeostasis were investigated in overweight and obese adolescent boys.

## Methods

### Study participants

Participants of this study included 108 male adolescents (15–17 year) from the Army Academy of Taiwan. Participants were classified as lean, overweight, or obese by comparing their BMI with age- and gender-appropriate cut-off values developed by the Department of Health of Taiwan [18]. Participants classified as overweight or obese were included in the overweight/obese group for analysis. Participants’ pubertal development was ≥IV, as assessed according to Tanner criteria [19] by a physician and/or a trained nurse practitioner. Exclusion criteria included the following: history of known heart disease, diabetes, renal disease, secondary obesity, or underlying genetic syndromes. All subjects had not participated in any weight loss program 6 months prior to the initiation of the present study, were not taking any medication, and were nonsmokers. The Institution Review Board of the Taipei Veterans General Hospital approved this study. Written informed consent was obtained after explaining the study procedures and protocol to the participants and their parents or legal guardians.

### Experimental control and pre-tests

All subjects were asked to record a baseline diet log before initiation of the study. Although education on the principles of a healthy diet was given and the participants were encouraged to decrease their food intake or at least consume a weight-maintaining high-carbohydrate diet, they chose their own food throughout the study.

All of the participants were also asked to refrain from vigorous exercise 3 days before the baseline anthropomorphic measurements. In addition to baseline measurements, all anthropomorphic measurements were taken again 7 days after completion of the 12-week exercise regimen in participants of the overweight/obese group. Anthropomorphic measurements were done between 8:00 and 8:30 AM following a 12-h fast; however, all participants were permitted to drink water. Each participant emptied his bladder and then height, weight and waist circumference (WC) were measured by trained staff. BMI was calculated as weight (in kilograms) divided by squared height (in square meters). The body fat percentage (BFP) and body fat mass (BFM) were measured using a bioelectrical body composition analyzer (Quantum X; RJL System, Clinton Township, MI, USA). An electrocardiogram was used to evaluate cardiac function in all subjects. Blood samples were taken from the antecubital vein of the arm at 9:00 AM. A 2-h oral glucose tolerance test (OGTT) was then performed according to a standardized procedure [20].

### Exercise regimen

Each participant in the overweight/obese group exercised five times per week, Monday through Friday, for 12 weeks. As part of the routine physical education training for the recruits, each 40-min session included a 10-min warm-up/flexibility period, a 25-min physical training period, and a 5-min cool-down period. During the warm-up/flexibility period, participants performed push-up and sit-up exercises followed by 5 min of stretching. In the physical training period, participants were asked to run moderately. This activity involved movement of the whole body to ensure maximum caloric expenditure. A high-intensity phase of physical training occurred in the last 5 min when participants were encouraged to run to the limit of their tolerance. In the cool-down period, the participants walked slowly for approximately 5 min. Throughout the exercise session, an experienced physical education instructor supervised all participants. Each week, a physician as well as participants’ legal guardians monitored one of the five sessions. In addition, all of the participants completed each session in the 12-week program, and compliance was 100%.

### OGTT

In the overweight/obese group, OGTT was performed 3 days prior to the exercise training period for the baseline assessments and 7 days following the end of the training program. Participants were asked to consume a weight-maintaining diet containing 250 g of carbohydrate per day and refrain from vigorous physical activity 3 days before the OGTT. An antecubital vein was cannulated for blood sampling at 8:50 AM. Baseline fasting blood samples were obtained at 9:00 AM after 10 min of rest. OGTT was performed with the administration of 75 g of anhydrate glucose in 300 mL of water within 5 min. Blood samples were drawn every 30 min for 2 h to evaluate plasma glucose and serum insulin levels.

The overweight/obese adolescents with impaired glucose tolerance (IGT) had impaired fasting glucose tolerance or glucose tolerance or both, as defined by American Diabetes Association recommendations [20].

### Laboratory measurements

Venous blood samples were taken after a 12-h fast for the measurement of fasting plasma glucose (FPG) and fasting serum insulin (FSI). After centrifugation, all sera were kept on ice immediately and stored at − 80 °C within 1 h. Plasma glucose was measured using the glucose oxidase method (Model 2300 STAT; Yellow Springs Instrument, Yellow Springs, OH, USA). Serum insulin was determined by a microparticle enzyme immunoassay using the AxSYM system from Abbott Diagnostics (Abbott Laboratories, Dainabot, Tokyo, Japan).

Homeostasis model assessment of insulin resistance (HOMA-IR) was calculated as follows: FSI × FPG/22.5, where insulin is expressed in μIU per mL and glucose in mmol/L) [21]. OGTT-derived parameters were used to evaluate insulin secretion [22]. Early-phase insulin release was calculated as the ratio of the change in insulin levels to the change in glucose levels from 0 to 30 min (∆I_0–30_/∆G_0–30_). Total insulin release was calculated using the ratio of insulin area under the curve (AUC) and glucose AUC during 0–120 min of the OGTT (InsAUC_120_/GluAUC_120_). AUC was calculated by the trapezoidal method from 0 to 120 min. Since the insulin response of β-cells to glucose is modulated by the severity of insulin resistance, the disposition index (DI), which adjusts insulin secretion for insulin resistance, was used to measure β-cell function [23] and was calculated as follows: (∆I_0–30_/∆G_0–30_)/HOMA-IR [24].

### Statistical analysis

Continuous data (i.e., height, weight, BMI, WC, hip circumference and waist-to-hip ratio) with normal distribution were expressed as mean ± standard deviation; data with non-normal distribution were expressed as median (with an interquartile range between P25 and P75). Differences in baseline characteristics and HOMA-IR between the lean group and group with obesity were examined by independent t-tests or Mann-Whitney U tests for parameters with normal or skewed distributions. Analysis of variance (ANOVA) was also performed to test differences in plasma glucose between the lean group and subgroups of the adolescents with obesity (i.e., those with normal or abnormal glucose tolerance). Bonferroni correction was carried out after a significant difference was revealed by ANOVA. The post-training changes were examined by paired t-tests for participants with obesity. A *P*-value < 0.05 was considered statistically significant. An adjusted alpha level of 0.017 (0.05/3) was applied when the Bonferroni correction was required. All statistical statistics were two-sided and performed using SPSS statistical software (version 22.0, IBM Corp., Armonk, NY).

## Results

### Exercise improved anthropomorphic parameters with exercise

The anthropometric parameters of the study participants, including 61 lean participants and 47 overweight/obese participants, are shown in Table 1. Before training, the height, weight, BMI, WC, hip circumference, BFP, BFM, and lean body mass of overweight/obese participants were significantly greater than those in the lean group (all *P* ≤ 0.037). These parameters remained greater in the overweight/obese group following training; however, significant improvements in all aforementioned parameters were observed in the overweight/obese group (all *P* ≤ 0.002). Specifically, their BMI, BFP and BFM decreased by 4.5, 28.2, and 30.3%, respectively. Moreover, the Matsuda/Defronzo index significantly increased in the overweight/obese participants following the exercise training (*P* < 0.001).

**Table 1:** Comparison of anthropometric parameters between overweight/obese male adolescents at baseline and after a 12-week exercise program with lean adolescents (*n* = 108)

	Lean *n* = 61)	Overweight/Obese before training (*n* = 47)	*P*_*baseline*_	Overweight/Obese after training (*n* = 47)	*P*_*after*_	*P*_*paired*_
Height (cm)	170.62 ± 5.79	172.61 ± 3.99	**0.037**	–	–	–
Weight (kg)	61.94 ± 5.93	83.21 ± 6.12	**< 0.001**	78.77 ± 7.6	**< 0.001**	**< 0.001**
BMI (kg/m^2^)	21.24 ± 1.31	27.9 ± 1.39	**< 0.001**	26.66 ± 2.12	**< 0.001**	**< 0.001**
Waist line (cm)	73.26 ± 4.53	90.61 ± 5.12	**< 0.001**	87.13 ± 5.36	**< 0.001**	**< 0.001**
Hip circumference (cm)	91.32 ± 3.65	105.78 ± 3.64	**< 0.001**	104.26 ± 4.54	**< 0.001**	**0.002**
W/H ratio	0.80 ± 0.03	0.86 ± 0.04	**< 0.001**	0.84 ± 0.03	**< 0.001**	**< 0.001**
BFP (%)	14.41 ± 4.36	25.14 ± 3.45	**< 0.001**	18.04 ± 5.06	**< 0.001**	**< 0.001**
BFM (kg)	9.06 ± 3.16	20.62 ± 3.95	**< 0.001**	14.37 ± 4.70	**< 0.001**	**< 0.001**
Lean mass (kg)	53.53 ± 4.74	62.36 ± 5.04	**< 0.001**	64.21 ± 5.52	**< 0.001**	**0.097**
Fasting plasma glucose (mmol/L)	4.37 ± 0.42	5.00 ± 0.41	**< 0.001**	4.58 ± 0.38	**0.008**	**< 0.001**
Fasting serum insulin (μIU/mL)	4.51 ± 2.15	7.58 ± 4.17	**< 0.001**	4.70 ± 2.32	0.676	**< 0.001**
Homeostasis model assessment of insulin resistance	0.74 (0.62, 1.08)	1.40 (0.93, 2.31)	**< 0.001**	0.86 (0.58, 1.36)	0.293	**< 0.001**
Total insulin release (InsAUC_0–120_) (μIU/mM)	–	4.65 (3.03, 6.01)	–	3.57 (2.84, 4.52)	–	**< 0.001**
Early-phase insulin release (μIU/mM)	–	8.62 (5.24, 12.25)	–	11.40 (6.76, 16.27)	–	**0.008**
Disposition index	–	5.84 (2.84, 9.67)	–	12.77 (7.27, 23.23)	–	**< 0.001**
Matsuda/Defronzo index	–	6.91 (1.87, 14.25)	–	12.55 (3.17, 22.54)		**< 0.001**

*BMI* body mass index, *BFP* body fat percentage, *BFM* body fat mass; *P*_baseline_ = *P*-values for group difference at baseline; *P*_after_ = *P*-values for group difference after training; *P*_paired_ = *P*-values of post-training changes in the overweight/obese group

All other data are shown as mean ± standard deviation

Bold values indicate a statistical difference between the two groups or between pre- and post-training, *P* < 0.05

Dash denotes ‘not applicable’

### Improved glucose homeostasis following a 12-week exercise program

As shown in Table 1, differences in FPG, FSI, and HOMA-IR were noted between the two groups at baseline. Specifically, the overweight/obese group had higher FPG, FSI and HOMA-IR (all *P* < 0.001). After the training program, significant decreases in FPG, FSI, HOMA-IR, and total insulin release as well as increases in early-phase insulin release and DI were observed in the overweight/obese group (all *P* ≤ 0.008).

**Fig. 1:**
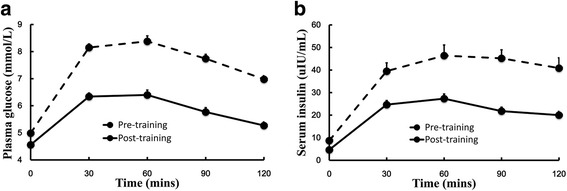
Effects of a 12-week exercise program on glucose and insulin levels in overweight/obese adolescents. Glucose (**a**) and insulin (**b**) levels obtained with an oral glucose tolerance test in 47 adolescents with obesity at baseline and after a 12-week exercise program. Data are shown as mean ± standard deviation. Pre- and post-training area under curves (AUCs) of plasma glucose and serum insulin were compared using paired t-tests for each group, and significant changes were found after the training (*P* < 0.001 for both plasma glucose and serum insulin)

Post-OGTT changes in plasma glucose and serum insulin in the overweight/obese group are illustrated in Fig. 1. Both parameters were significantly diminished after the training (Fig. 1a & b).

The overweight/obese group was further divided into two subgroups based on their pre-training glucose homeostasis status, including 36 with normal glucose tolerance (NGT) and 11 with impaired glucose tolerance (IGT; Table 2). As shown in Table 2, both groups of adolescents had common anthropometric measures, including weight, BMI, WC, hip circumference, W/H ratio, and lean body mass. Moreover, no differences in total cholesterol, low-density lipoprotein-cholesterol, high-density lipoprotein–cholesterol, alanine aminotransferase (U/L), and uric acid were noted (data not shown).

**Table 2:** Anthropometric parameters in overweight/obese adolescents and normal or impaired glucose tolerance before training (*n* = 47)

	NGT (*n* = 36)	IGT (*N* = 11)	*P*-value
Height (cm)	172.44 ± 3.88	173.18 ± 4.47	0.593
Weight (kg)	82.71 ± 4.98	84.86 ± 9.02	0.463
Body mass index (kg/m^2^)	27.8 ± 1.15	28.24 ± 2.04	0.508
Waist circumference (cm)	90.43 ± 4.23	91.23 ± 7.56	0.743
Hip circumference (cm)	105.29 ± 3.28	107.36 ± 4.43	0.099
W/H ratio	0.86 ± 0.04	0.85 ± 0.05	0.479
Body fat percentage (%)	24.00 (22.00, 26.00)	28.00 (26.00, 29.00)	**0.038**
Body fat mass (kg)	19.70 (18.00, 22.40)	22.55 (20.00, 27.10)	**0.047**
Lean mass (kg)	62.41 (50.54, 72.51)	62.13 (53.38–72.88)	**0.906**

Height, weight, body mass index, waist, hip and W/H ratio are shown as mean ± standard deviation, and the other parameters are expressed as median (interquartile range)

Bold values indicate a statistical difference between the two groups, *P* < 0.05

Although overweight/obese patients in the IGT group had higher BFP and BFM before training, the values in the IGT group reduced to the level similar to those in the NGT group (*P* = 0.066 and *P* = 0.090, respectively) after the exercise program (data not shown). In terms of indices of homeostasis, the IGT group had higher baseline FPG, FSI, HOMA-IR, total insulin release, and lower DI than the NGT group (all *P* ≤ 0.039; Table 3). This group also had lower Matsuda/Defronzo index score (*P* < 0.001) After the exercise training program, significant reductions in FPG, FSI, HOMA-IR, and total insulin release (all *P* ≤ 0.034), along with an increase in the DI (both *P* ≤ 0.011) were observed in both groups (Table 3). In the NGT group, the level of insulin release was also significantly increased (*P* = 0.025). Compared with the NGT group, patients in the IGT group had greater changes in FPG, FSI, HOMA-IR, and total insulin release, and the pre-training differences between two groups were no longer significant. No differences in the Matsuda/Defronzo index values between the NGT and IGT groups were observed following the exercise program (Table 3).

**Table 3:** Homeostasis parameters in overweight/obese adolescents and normal or impaired glucose tolerance after training (*n* = 47)

	NGT before training (*n* = 36)	IGT before training (*n* = 11)	*P*_*before*_	NGT after training (*n* = 36)	IGT after training (*n* = 11)	*P*_*after*_
Fasting plasma glucose (mmol/L)	4.83 (4.61, 5.14)	5.39 (5.11, 5.72)	**< 0.001**	4.55 (4.4, 4.77)	4.61 (4.47, 4.96)	0.374
Fasting serum insulin (μIU/mL)	5.6 (4.4, 8.9)	9.15 (8.1, 12.1)	**0.009**	4.5 (3, 6.5)	4.2 (2.8, 4.70)	0.395
HOMA-IR	1.29 (0.88, 1.92)	2.28 (1.84, 2.9)	**0.004**	0.87 (0.59, 1.36)	0.86 (0.55, 0.96)	0.520
Total insulin release (μIU/mM)	4.04(3.01, 5.55)	6.52 (4.39, 9.11)	**0.039**	3.57 (3, 4.36)	3.95(2.58, 6.06)	0.479
Early-phase insulin release (μIU/mM)	8.49(5.44, 11.72)	10.22 (1.55, 19.77)	0.951	11.16 (6.46, 15.53)	12.83(7.34, 20.17)	0.209
Disposition index	6.71(3.43, 12.79)	3.19 (0.95, 6.82)	**0.032**	12.46 (6.11, 23.52)	14.90(9.93, 22.70)	0.366
Matsuda/Defronzo index	7.58 (2.76–14.58)	4.08 (1.87–7.32)	**< 0.001**	12.97 (5.32, 22.54)	10.75 (3.17, 18.00)	0.245
*Change from baseline*						***P***_***group***_
Fasting plasma glucose (mmol/L)	–	–	–	−0.40 (−0.53, −0.07)*	−0.64 (− 0.94, − 0.32)*	**0.018**
Fasting serum insulin (μIU/mL)	–	–	–	−1.50 (−3.00, 0.00)*	−6.15 (−8.20, −2.80)*	**0.007**
HOMA-IR	–	–	–	− 0.48 (− 0.76, 0.02)*	−1.50 (− 2.01, − 0.68)*	**0.005**
Total insulin release (μIU/mM)	–	–	–	− 0.49 (− 1.67, 0.43)*	−1.92 (− 3.21, − 0.99)*	**0.034**
Early-phase insulin release (μIU/mM)	–	–	–	2.25 (− 0.22, 6.30)*	2.12 (1.33, 14.90)	0.482
Disposition index	–	–	–	5.29 (− 0.83, 15.18)*	13.07 (4.11, 22.41)*	0.074
Matsuda/Defronzo index				5.39 (−5.72, 18.71)*	6.67 (0.53, 13.50)*	0.456

*HOMA-IR* homeostasis model assessment of insulin resistance; *P*_before_ = *P*-values for group differences before training; *P*_after_ = *P*-values for group differences after training; *P*_group_ = *P*-values for group differences in changes from baseline

Data are presented as median (interquartile range)

Dash indicate data are unavailable

* denotes significant change after training

Bold values indicate a statistical difference between the two groups or a significant change after training, *P* < 0.05

**Fig. 2:**
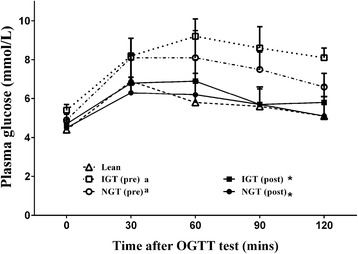
Change in plasma glucose levels in a 2-h oral glucose tolerance test (*n* = 61 in the lean group marked in black diamond, *n* = 36 in the NGT group marked in white circle for pre-training and black circle for post-training, and *n* = 11 in the IGT group marked in white square for pre-training and black square for post-training). Mean ± standard deviation is shown for plasma glucose. Area under curve (AUC) of plasma glucose was used for comparisons among the groups. Analysis of variance was implemented for testing the difference in plasma glucose among the three groups (i.e. the lean, the IGT and NGT groups), and paired tests were employed for testing post-training differences for the two subgroups. ^*^ significant change after training. ^a^ significant difference between either the IGT or NGT group and the lean group

Changes in plasma glucose levels in the IGT and NGT subgroups of overweight/obese adolescents are shown in Fig. 2. Before training, the plasma glucose level was significantly higher in both NGT and IGT groups than in the lean group (*P* < 0.001). However, after training, there was no difference in plasma glucose among the three groups.

## Discussion

Given the increase in childhood obesity and associated metabolic abnormalities and their link with chronic disease in adults, interventions are urgently needed. The present study evaluated the effects of exercise training without diet control in male adolescents who were overweight or obese. The major finding of the present study is that 12 weeks of exercise training alone can improve insulin secretion in addition to the improvement in insulin sensitivity and decrease in body mass, suggesting that this intervention is effective to induce glucose homeostasis in adolescent obesity. Although significant changes in BW and BMI in the overweight/obese adolescents were about 5.3 and 4.4%, respectively, following exercise training, their BFP and BFM values declined about 28.2 and 30.3%, respectively, suggesting that the loss in body mass was predominantly composed of fat. Furthermore, the insulin sensitivity of the overweight/obese group significantly increased following exercise training even in patients with abnormal glucose tolerance at baseline (i.e., the IGT group).

Impaired insulin sensitivity, which may appear prior to glucose dysregulation in adolescents [25], is a major component of obesity and its co-morbid diseases, such as T2DM and cardiovascular disease (CVD). However, studies evaluating the impact of exercise on insulin sensitivity in children and adolescents with obesity have been inconsistent. In prepubertal girls with obesity, both insulin sensitivity and intra-abdominal adipose tissue remained unchanged following a strength-training program [26]. In another study, strength exercise did not change peripheral insulin sensitivity but improved the muscle mass and hepatic insulin sensitivity in youth with obesity [27]. However, other studies showed that physical training improves insulin sensitivity despite no change in fat mass [28, 29]. In the present study, most of the participants in the overweight/obese group showed significant reductions in body mass and fat mass, and their FSI and HOMA-IR were reduced by about 38, and 39%, respectively. In contrast, Abrams et al. [30] reported that a decrease in BMI of 8% was required for improvements in insulin sensitivity in adolescents participating in a 4-month behavioral weight loss trial.

The conflicting data regarding the effects of exercise on anthropomorphic measures and insulin sensitivity are likely due to differences in the exercise modality. For example, postpubertal adolescents participating in a combined aerobic and resistance training program experienced greater losses of body fat and WC [31]. Furthermore, in a randomized controlled trial comparing resistance versus aerobic exercise in adolescent boys with obesity, improvements in insulin sensitivity were observed with resistance exercise alone [17].

Subjects with obesity frequently have basal hyperinsulinemia and an exaggerated response to stimulation by a test meal or glucose attributed to increased insulin secretion and reduced insulin clearance [32, 33]. In the present study, both the FSI and total insulin response during OGTT decreased significantly after the exercise training, which is in agreement with previous studies showing improved fasting and stimulatory insulin response after weight loss [34–36].

Previous work has highlighted the beneficial effects of lifestyle interventions on insulin secretion and β-cell function in adults with obesity [11–13]; however, few adolescent-based studies have been conducted to date [17]. In addition, a defect in first-phase insulin response is an early event in the development of T2DM in youth [6, 7], and decreased β-cell function in Latino adolescents with overweight has been noted after Tanner 3 [37]. In the present study, we found significant increase in the DI after the 12-week training, suggesting an improvement in β-cell function. This finding is promising because it suggests that impairment of first-phase insulin secretion is reversible at least in part. This notion is further supported by the OGTT results, which showed significant decreases in both glucose and insulin levels at all the time points analyzed.

It has been previously reported that both strength training and aerobic exercise improve hepatic insulin sensitivity [26, 27], and our HOMA-IR results further support this notion. However, HOMA-IR and OGTT are rough indices of whole body insulin sensitivity and hepatic insulin sensitivity. Matsuda et al. [38] described a simple, novel estimate of whole body and hepatic insulin sensitivity using indices derived from the OGTT, which correlated well with values obtained with the euglycemic insulin clamp. Thus, further studies will examine whether the impact of this particular exercise program was specific to improving hepatic insulin sensitivity.

The present study is limited in that it included only Taiwanese male adolescents. In addition, the sample sizes of both groups were relatively small, and the OGTT was only performed in the participants with obesity. Therefore, the result may not be applicable to female adolescents or populations of different ethnicity, which is particularly relevant given the ethnic differences in first-phase insulin secretion observed among peripubertal children [39]. Additional studies are also necessary to examine if a longer exercise intervention would provide further improvements in insulin sensitivity and anthropomorphic measures. Furthermore, measures of exercise intensity and energy expenditure (i.e., heart rate, accelerometer or pedometer measures, or measures of perceived exertion) were not determined. Finally, although the participants were encouraged to decrease their food intake during the study period, measures of food intake or food records were not maintained.

## Conclusions

In conclusion, a 12-week exercise training program significantly improved the insulin sensitivity and insulin secretion in overweight or obese male adolescents even in those with impaired glucose tolerance. These improvements were associated with reductions in both fasting and 2-h glucose levels. Thus, exercise training may represent a critical intervention for preventing not only diabetes, but also future CVD. Further studies with larger sample sizes and longer training periods are warranted.

## Abbreviations

ANOVA: Analysis of variance
AUC: Area under the curve
BFM: Body fat mass
BFP: Body fat percentage
BMI: Body mass index
CVD: Cardiovascular disease
DI: Disposition index
FPG: Fasting plasma glucose
FSI: Fasting serum insulin
HOMA-IR: Homeostasis model assessment of insulin resistance
IGT: Impaired glucose tolerance
Il-6: Interleukin-6
NGT: Normal glucose tolerance
OGTT: Oral glucose tolerance test
T2DM: Type 2 diabetes mellitus
WC: Waist circumference
